# Kisspeptin mediation of estradiol-induced secretion of luteinizing hormone and prolactin

**DOI:** 10.1186/1471-2202-15-S1-P147

**Published:** 2014-07-21

**Authors:** Natalia Toporikova, Philip Dishuck, Joel Tabak, Cleyde Helena

**Affiliations:** 1 Biology Department, Washington and Lee University, Lexington, VA, 24450, USA; 2 Program in Neuroscience, Florida State University, Tallahassee, FL, 32306, USA

## 

In females, ovulation is coordinated by a complex set of interactions between the hypothalamus, the anterior pituitary and the gonads. Luteinizing hormone (LH) and prolactin (PRL) are pituitary hormones that have complementary roles in reproduction. There is a surge of both hormones prior to ovulation that is essential for adequate reproduction and can be mimicked by estradiol (E2) treatment in ovariectomized (OVX) rats [1], [2]. Both the preovulatory and the E2-induced LH and PRL surges in OVX rats occur at the same time of the day, indicating that circadian signals originating in the suprachiasmatic nucleus (SCN) must provide input to coordinate both LH and PRL secretion, and that response to this input depends on the steroid *milieu*. Kisspeptin (KISS) is the most potent stimulator of LH release and it has been recently demonstrated to also stimulate PRL release. There are two KISS neuronal populations in the rodent brain, located in the anteroventral periventricular nucleus (AVPV) and the arcuate nucleus (ARC). The ARC population coexpress kisspeptin, neurokinin B and dynorphin and are called KNDy neurons.

**Figure 1:**
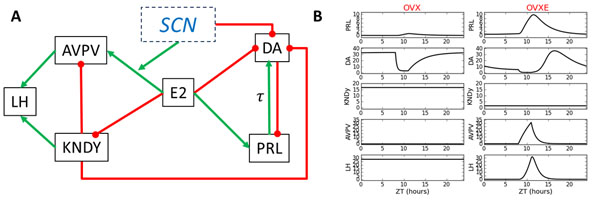
A mathematical model reproducing the surges of LH and PRL. (A) Model diagram which was implemented in a system of differential equations. (B) Results of modeling simulations for OVX (left) and OVXE (right). Levels of hormones and neurotransmitters (in arbitrary units) are plotted on the y-axis. ZT is circadian time, where ZT0 represent the time of lights-on.

We have developed a reduced model for kisspeptin control of PRL and LH secretion in animals without (OVX) and with E2 treatment (OVXE), based on experimental data and literature describing the roles of the two kisspeptin subpopulations (KNDy and AVPV) in PRL and LH secretion (Fig 1A). Without E2, secretion of PRL and LH remains constant. Dopamine (DA) is released from E2 inhibition and tonically inhibits PRL secretion [3]. The absence of E2 inhibition to KNDy neurons stimulates a tonic increase of LH secretion, while the AVPV subpopulation has a low level of activity, thus preventing an LH surge (Fig 1B, left). In the OVXE model, E2 directly induces a PRL surge by increasing the activity of lactotrophs and indirectly inhibiting DA neurons (Fig 1B, right). The time-of-day-signal is provided by a brief E2-dependent stimulation from neurons in the SCN. E2 inhibition of KNDy neurons decreases LH basal secretion while stimulation of the AVPV by E2, together with the circadian SCN signal, produces the LH surge (Fig 1B, right). The modeling simulation predicts that the two KISS subpopulations have dramatically different effects on the LH surge: ablation of AVPV eliminates the surge without changing basal LH levels, while ablation of KNDY neurons would reduce basal levels and have a modest effect on the LH surge. KNDy ablation would also reduce the PRL surge.
